# Isolation of a Dissimilatory Iodate-Reducing *Aromatoleum sp.* From a Freshwater Creek in the San Francisco Bay Area

**DOI:** 10.3389/fmicb.2021.804181

**Published:** 2022-01-17

**Authors:** Victor Reyes-Umana, Jessica Kretschmer, John D. Coates

**Affiliations:** Department of Plant and Microbial Biology, University of California, Berkeley, Berkeley, CA, United States

**Keywords:** iodate, iodide, *Aromatoleum*, anaerobic, genomic island, iodine

## Abstract

Recent reports of dissimilatory iodate-reducing microorganisms (DIRM) have arisen from studies of bacteria in marine environments. These studies described the physiology and distribution of DIRM while also demonstrating their presence in iodine-rich marine environments. We posited that despite lower iodine concentrations, terrestrial and freshwater ecosystems should also harbor DIRM. We established numerous enrichments from coastal and freshwater environments that actively remove amended iodate. We describe the physiology and genome of a new DIRM isolate, *Aromatoleum toluclasticum* sp. TC-10, emerging from a freshwater creek microcosm. Like other DIRM, *A. toluclasticum* sp. TC-10 couples acetate oxidation to iodate reduction with a concomitant increase in the OD_600_. Our results indicate that *A. toluclasticum* sp. TC-10 performs dissimilatory iodate reduction (DIR) using the recently described iodate reductase (Idr). We provide further evidence of horizontal gene transfer of the *idr* genes by demonstrating the lack of Idr in the closely related (99.93% 16S rDNA sequence identity) *A. toluclasticum* sp. MF63 and describe the heterogeneity of the accessory proteins associated with the iodate reduction island (IRI). These observations provide additional evidence that DIR is a horizontally acquired metabolism with broad environmental distribution beyond exclusively marine environments.

## Introduction

Iodine (as ^127^I) is an essential component of the mammalian diet for its role in thyroxine biosynthesis. The element is abundant in seawater, where it exists at several oxidation states, averaging concentrations of about 450 nM (Küpper et al., 2011). Kelp and algae bioconcentrate iodine as iodide (I^–^) and produce iodocarbons that ultimately impact atmospheric chemistry and climate by catalyzing tropospheric ozone destruction (Manley and Dastoor, 1988; Cuevas et al., 2018). In addition to a marine biogeochemical cycle, iodine also exhibits a terrestrial phase. Wet deposition of iodine in rainwater is a common way for iodine to enter terrestrial ecosystems, with rainwater concentrations ranging from 4 to 47 nM (Fuge and Johnson, 2015). Dry deposition similarly relies upon iodine evolution from marine sources but is delivered through volatilized iodocarbons and iodine to littoral environments (Saiz-Lopez et al., 2012). A higher iodine flux occurs by dry deposition (3.6–6.5 μmol m^–2^ yr^–1^) than wet deposition (2.7 μmol m^–2^ yr^–1^) (Baker et al., 2001). Ultimately, iodine deposition depends on distance from the ocean, with low soil iodine concentrations (<5 ppm) found at locations beyond 70 kilometers (Fuge and Johnson, 2015).

As the importance of the iodine biogeochemical cycle on both human health and global climate emerges, the biological mechanism behind the unexpected disequilibrium between iodate (IO_3_^–^) and I^–^ remains an open question (Luther et al., 1995). The physiochemical state of water suggests that iodine is most stable as IO_3_^–^, yet I^–^ predominates across many regions of high biological productivity globally (Luther et al., 1995; Gonzales et al., 2017). Recent work suggests that a diverse group of dissimilatory iodate reducing microorganisms (DIRM) consisting mainly of marine *Proteobacteria* may contribute to this phenomenon in oceanic environments (Reyes-Umana et al., 2021). Metagenomic surveys indicate that DIRM are ubiquitous in the Earth’s oceans and are associated with marine life or live above oxygen minimum zones (Reyes-Umana et al., 2021). DIRM are defined characteristically by the ability to reduce relatively high concentrations of iodate (IO_3_^–^) to iodide (I^–^) and couple it to growth. Dissimilatory iodate respiration (DIR) is dependent on a dedicated iodate reduction genomic island (IRI) that is horizontally transferred between diverse taxa (Reyes-Umana et al., 2021). The IRI is composed of a heterodimeric iodate reductase (IdrAB), and two distinct putative cytochrome c peroxidases (IdrP1 and IdrP2). The IdrA is a highly conserved molybdenum-dependent member of the DMSO reductase protein superfamily closely related to dissimilatory nitrate reductases and perchlorate reductases (Reyes-Umana et al., 2021).

Little is known about DIR in terrestrial or freshwater environments. However, the presence of iodine in many freshwater systems at variable oxidation states (Baker et al., 2001; Fuge and Johnson, 2015) suggests the existence of a microbially mediated terrestrial iodine redox cycle. Such an observation would be consistent with metabolisms like dissimilatory perchlorate and chlorate respiration that show broad distribution in environments with variable chlorate and perchlorate concentrations (Coates et al., 1999). In this study, we collected sediment from numerous freshwater environments throughout the San Francisco Bay Area and cultured bacteria capable of reducing iodate to iodide. We describe the isolation of a new strain of *Aromatoleum toluclasticum*, TC-10, from a freshwater environment, containing low chloride concentrations (∼70 mg/L Cl^–^) (Farby, 2020) and no marine water input. Like TC-10, the *Aromatoleum* type strain, *A. toluclasticum* sp. MF63, was also isolated from a freshwater environment (Song et al., 1999). The *Aromatoleum* genus is widely distributed in the environment and displays significant eco-physiological diversity potentiated by its large pangenome and numerous mobile elements (Martín-Moldes et al., 2015; Rabus et al., 2019). Consistent with that, we show that TC-10 is a DIRM that possesses the *idrABP_1_P_2_* genes, enabling it to grow *via* dissimilatory iodate reduction, making it the first freshwater DIRM isolated. We compare the genome of TC-10 to the type strain *Aromatoleum toluclasticum* sp. MF-63 (Song et al., 1999), and show the iodate reducing capability is specific to TC-10 and mobile in *Aromatoleum*. These results ultimately broaden the range to search for novel DIRM and provide a possible ecological role for free-living *Aromatoleum* species beyond their ability to degrade petroleum-based contaminants (Reinhold-Hurek et al., 2015).

## Results

### Isolation of *Aromatoleum toluclasticum* sp. TC-10

**FIGURE 1:**
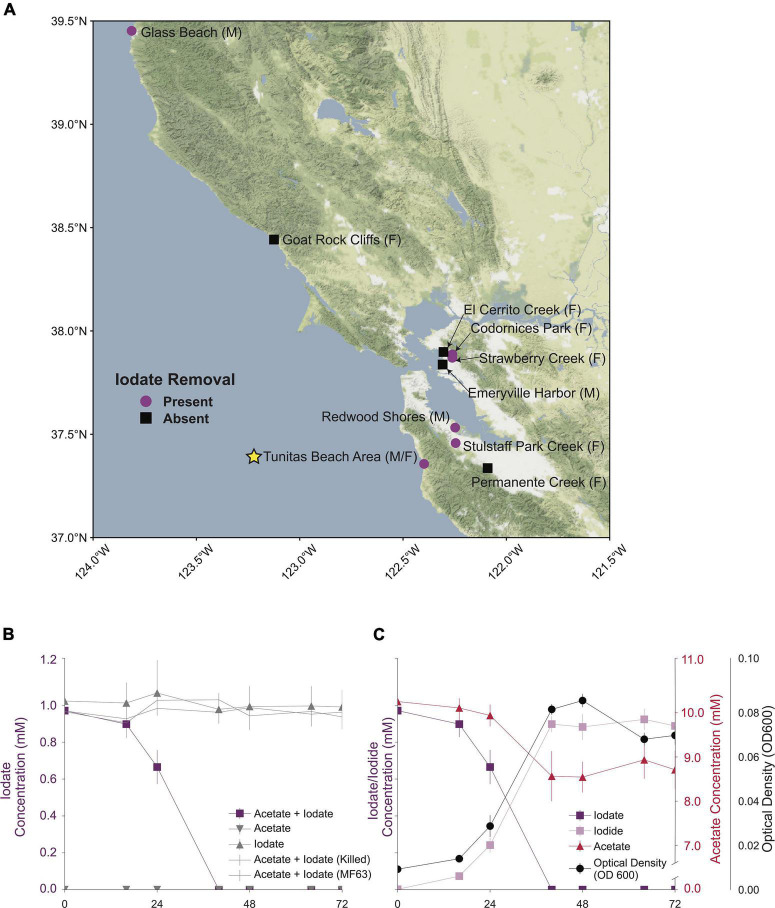
**(A)** A map of the San Francisco Bay Area showing all sampled locations. Locations sampled for marine enrichments denoted by (M) and locations sampled for freshwater enrichments denoted by (F) Samples from locations showing removal of 2 mM iodate denoted by purple circles (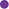). The location where *Aromatoleum toluclasticum* sp. TC-10. A yellow star denotes TC-10. Samples from locations without iodate removal are denoted by black boxes (■). **(B)** A time course showing removal of iodate in the acetate + iodate condition and no removal of iodate in alternative conditions. **(C)** A time course showing the removal of acetate, iodate, and the production of iodide, coupled to an increase in the optical density of the cell culture.

Aquatic sediments from around the San Francisco Bay Area were collected as inoculum for ten sets of anoxic enrichment cultures, each amended with 10 mM acetate and 2 mM iodate. Complete iodate removal after 14 days was observed in six out of ten microcosm sets (Figure 1A). Active microcosms were passaged three times in anoxic basal media and subsequently grown on R2A agar plates aerobically. A single colony from the Tunitas creek microcosm (San Gregorio, California) yielded a non-fastidious culturable isolate. 16S rRNA gene sequencing indicated an axenic culture closely related (99.93% sequence identity) to *Aromatoleum toluclasticum* sp. MF63^T^ (ATCC 700605), in the family *Rhodocyclaceae* of the phylum *Proteobacteria*. *A. toluclasticum* sp. MF63 was initially isolated by Song et al. (1999) in Moffet Field, California, about 34 miles east of San Gregorio, from shallow aquifer sediment in 1999. To differentiate our isolate from MF63, we designated the latest strain as TC-10, referring to “Tunitas creek” and its microcosm bottle number. Like MF63, TC-10 forms short motile rods and is a non-fastidious facultative anaerobe. Anaerobically, TC-10 grows on acetate or lactate with nitrate or iodate as sole electron acceptors. The genus has recently been reclassified as *Aromatoleum* in response to emerging biological and comparative genomic data (Rabus et al., 2019). Interestingly, both *A. toluclasticum* sp. MF-63 and TC-10 possess nearly identical NarGHI proteins (>99% amino acid identity); however, in contrast to strain TC-10, strain MF-63 cannot utilize IO_3_^–^, supporting previous observations that the dissimilatory nitrate reductase does not enable iodate reduction in DIRM (Amachi et al., 2007; Reyes-Umana et al., 2021).

### Dissimilatory Iodate Reduction in *Aromatoleum toluclasticum* sp. TC-10

Cells of *A. toluclasticum* sp. TC-10 grew on a minimal freshwater medium with acetate and IO_3_^–^ as the sole electron donor and acceptor, respectively. The working IO_3_^–^ concentration was reduced from 2 mM to 1 mM, shortening the lag phase in TC-10. Growth studies revealed that TC-10 accumulated cell mass as measured by an increase in optical density at 600 nm (OD_600_) with concomitant reduction of IO_3_^–^ to I^–^ (Figure 1B). No optical density increase occurred for heat-killed cultures or under conditions lacking either acetate or iodate. Ion chromatography data showed that TC-10 consumed a total of 0.98 ± 0.05 mM IO_3_^–^ (mean ± standard deviation, *n* = 4) while oxidizing 1.31 ± 0.25 mM acetate (mean ± standard deviation, *n* = 4), resulting in a final optical density increase of 0.076 (Figures 1B,C). These data suggest an average stoichiometry of 0.75 mol of IO_3_^–^ consumed per mol of acetate. Optical density to dry weight relationships for rod-shaped bacteria of similar dimensions (*E. coli*) suggests that an OD_600_ increase of 1.0 is equivalent to 0.39 grams dry cell weight per liter (Glazyrina et al., 2010). Assuming a dry cell mass of 50% carbon, the corrected stoichiometry accounting for acetate incorporation into cells is 90% of the theoretical value according to:


(1) $$3\,{\mathrm{CH}}_{3}\,\text{COOH}+4\,{\text{IO}}_{3}^{-}\to 6\,{\mathrm{CO}}_{2}+4\,{\text{I}}^{-}+6\,{\text{H}}_{2}\,\text{O}$$

The stoichiometry, energetics, and mechanism of iodate reduction are similar in both *A. toluclasticum* sp. TC-10 and the previously described DIRM *Denitromonas* sp. IR-12 (Reyes-Umana et al., 2021). In *A. toluclasticum*, 52% of the total carbon is assimilated into biomass, whereas *Denitromonas* sp. IR-12 assimilated 31% of total carbon into biomass. Moreover, the doubling time for TC-10 growing on iodate (T_D_ = 7.7 h, μ = 0.09) is on the same order of magnitude as *Denitromonas* sp. IR-12 (T_D_ = 11.0 h, μ = 0.06). These results are consistent with biomass accumulation patterns in organisms using highly oxidized electron acceptors like nitrate (NO_3_^–^/N_2_, *E^o^*′ = + 0.713 V) (Weber et al., 2006) and perchlorate (ClO_4_^–^/Cl^–^, *E^o^*′ = + 0.797 V) (Youngblut et al., 2016). Furthermore, while iodate is a suitable terminal electron acceptor for either organism (IO_3_^–^/I^–^ *E^o^*′ = + 0.72 V), both *A. toluclasticum* sp. TC-10 and *Denitromonas* sp. IR-12 grow faster on O_2_ (T_D_ = 1.23 h-1.64 h, μ = 0.42–0.56), which is consistent with oxygen’s energetic favorability (O_2_/H_2_O, *E^o^*′ = + 0.820 V).

**FIGURE 2:**
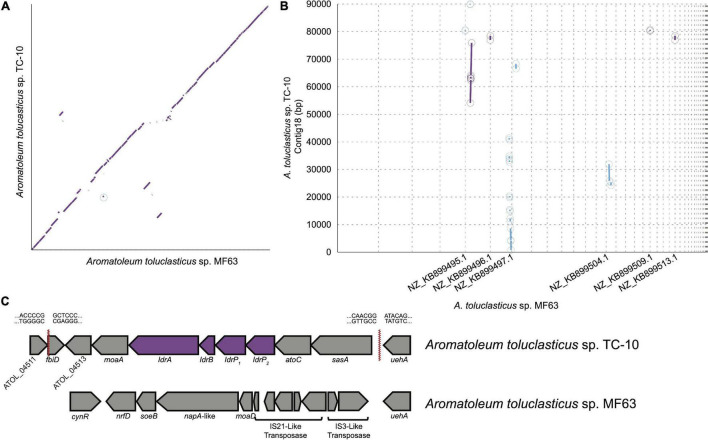
**(A)** A synteny plot showing regions of major synteny, weak synteny, and gaps between *Aromatoleum toluclasticum* MF63 and TC-10. **(B)** A synteny plot showing the regions of conservation along node 18 in TC-10, which contains the IRI. The gap between 41,363 and 54,119 bp signifies the region containing the IRI. **(C)** The IRI in TC-10 with catalytic *idr* genes is denoted by the purple color. Red serrated lines denote borders of the IRI. Sequences flanking serrated lines represent the nucleotide sequence at either side of the IRI border. MF63 is provided below as a comparison to demonstrate the lack of the IRI near *uehA*.

Prior examples of DIRM show that they possess a genomic island known as the IRI (Reyes-Umana et al., 2021). This island contains *idrABP_1_P_2_* genes that enable DIRM to respire IO_3_^–^ as a terminal electron acceptor (Reyes-Umana et al., 2021). Like the DIRM *Denitromonas* sp. IR-12 and *Pseudomonas* sp. SCT, *A. toluclasticum* sp. TC-10 possesses the IRI, which is lacking in the closely related, non-iodate reducing, *A. toluclasticum* sp. MF-63 (Figure 2C). This observation is consistent with recent evidence demonstrating the necessity of *idrA* for iodate respiration in *Denitromonas* sp. 1R-12 (Reyes-Umana et al., 2021). The genome of *A. toluclasticum* sp. TC-10 also provides additional evidence consistent with the proposed hybrid enzymatic-abiotic model proposed for DIR in *Denitromonas* sp. 1R-12 (Reyes-Umana et al., 2021). In this model, iodate is initially reduced by IdrAB which accepts electrons from the quinone pool *via* a cytochrome c551 (cyt c551) and performs a four-electron transfer with a resultant production of the chemically unstable intermediate hypoiodous acid (HIO). The HIO then undergoes abiotic disproportionation to yield I^–^ and IO_3_^–^ in a 2:1 ratio. Both TC-10 and MF63 lack genes resembling chlorite dismutase (*cld*), further supporting that *cld* is not involved in DIR as was proposed for *Pseudomonas* sp. SCT (Yamazaki et al., 2020). Apart from *Pseudomonas* sp. SCT and a *Sedimenticola thiotaurini* from a marine metagenome, *idrA* and *cld* genes are mutually exclusive. The lack of *cld* in TC-10 is consistent with the observed mutual exclusivity of organisms with *cld* and *idr* (Reyes-Umana et al., 2021). Together, these observations support the parsimonious DIR model involving a specialized iodate reductase that performs a four-electron transfer to an unstable hypoiodous acid intermediate that disproportionates to IO_3_^–^ and I^–^ (Reyes-Umana et al., 2021).

### Evolutionary History of the Iodate Reduction Island in *Aromatoleum toluclasticum* sp. TC-10

Both strains of *Aromatoleum toluclasticum* share a close evolutionary history given the high degree of similarity between both organisms, presenting a unique opportunity to study the IRI. 16S rRNA gene sequencing shows 99.93% nucleotide identity for both *Aromatoleum toluclasticum* strains between positions 27 and 1492. Whole-genome sequencing of TC-10 shows an average nucleotide identity of 98.20% to the type strain *Aromatoleum toluclasticum* sp. MF63, affirming the 16S phylogeny of TC-10. Since DIR is a distinguishing feature of TC-10, we generated a synteny plot to show the conserved regions, rearrangement, and insertion between both strains (Figure 2A). The synteny plot identified an 89 kb contig in strain TC-10 with numerous inconsistent gaps mapping to the MF63 genome (Figure 2B). In TC-10, the *idr* genes are located on a 12.7 kb region directly upstream from a stretch of identical sequence near the *ueh* operon. The island is flanked further by a short segment mapping to an arsenosugar biosynthesis gene *fbiD* (TIGR04282) in the MF63 genome (Figure 2C). The discontinuity occurs at an intergenic region at the 3′ end and within the *fbiD* gene at the 5′ end. The TC-10 genome assembly shows the IRI in the middle of a long contig; however, the flanking regions map to two separate contigs on the MF63 genome, suggesting genome rearrangement has occurred. The same region in MF63 lacks *idr* genes but has an IS3-like transposase and associated hypothetical proteins. While the same IS3-like transposase is present in TC-10, the IS element is not associated with the IRI in its genome.

Traditional markers of horizontal gene transfer such as GC skew or inverted repeats were absent at the site of IRI integration in TC-10. GC content at the IRI is similar to the genome at large (64.75% GC island vs. 66.08% GC genome), and tools such as Tandem Repeats Finder (Benson, 1999) identified no tandem repeats indicative of transposase mediated insertion. Further investigation warranted the use of tools with more robust predictive capacity, such as IslandViewer4 (Bertelli et al., 2017) and MGEfinder (Durrant et al., 2020). The latter method identified numerous mobile genetic elements using both its standard and sensitive modes but did not identify regions surrounding the IRI as a mobile genetic element. Similarly, IslandViewer4 did not identify the IRI as a mobile genetic element; however, a 4.6 kb island containing the molybdopterin biosynthesis gene *moaA* and the arsenosugar biosynthesis gene *fbiD* was predicted as mobile by codon usage bias with an HMM (SIGI-HMM) (Bertelli et al., 2017). Since the IRI starts at *fbiD* in TC-10, it is possible that mechanisms mobilizing these genes also mobilize *idr*, and similar codon usage bias in known DIRM at the *idr* locus obfuscates its provenance. The evolutionary history of the *idr* gene neighborhood in TC-10 provides further evidence for horizontal transfer. To date, all experimentally confirmed DIRM belong to a small clade with ten genomes with diverse amino acid primary sequences—the amino acid identity (AAI) between TC-10 and *Denitromonas* sp. IR-12 IdrA is 76%, and 84% between TC-10 and *Pseudomonas* sp. SCT. There is also significant conservation of synteny between *idrABP_1_P_2_*, consistent with the previous observations (Reyes-Umana et al., 2021). The gene product for *idrAB*, a prerequisite for DIR, forms a phylogenetically similar molybdopterin oxidoreductase to arsenite oxidase (Aio; Yamazaki et al., 2020; Reyes-Umana et al., 2021). The *idrAB* is always preceded by two putative cytochrome c peroxidases *idrP_1_P_2_*, whose products share only 37% AAI, suggesting that both play separate roles in detoxification reactions (Reyes-Umana et al., 2021). Taken together, synteny and predicted gene function suggest that *idrABP_1_P_2_* have similar roles in both *A. toluclasticum* and other DIRM.

**FIGURE 3:**
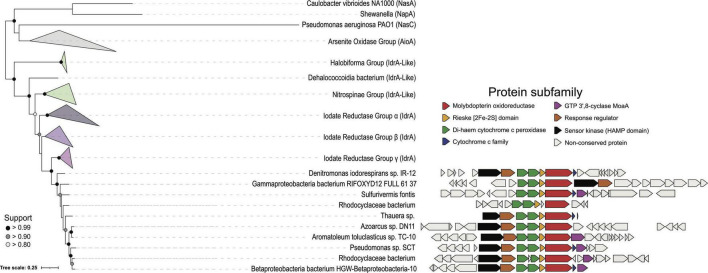
A phylogenetic tree representing the major groups of IdrA. Collapsed clades are represented as triangles to show the interclade distance. IdrA clade containing all cultured representatives of DIRM is expanded. Gene neighborhoods show ± 10 genes from IdrA when present. Genes are colored based on Pfam annotation if the protein family shows up in more than 50% of the clade’s genomes.

Outside of the *idr* genes, several putative DIRM genomes that clade with known DIRM are missing accessory genes sometimes associated with IRI. Out of the ten genomes, two are missing the two-component system, five are missing a c551-like cytochrome c, and five are missing the *moaA* gene (Figure 3). The two-component system likely plays a role in sensing IO_3_^–^ in the environment, as Idr expression has been demonstrated to be inducible upon exposure to IO_3_^–^ (Amachi et al., 2007; Yamazaki et al., 2020). The cytochrome c551 is a soluble electron transfer protein that presumably plays a role in transferring electrons from the quinone pool *via bc1* (Reyes-Umana et al., 2021). However, given its prevalence, organisms without cyt c_551_ in the IRI likely use a cyt c_551_ encoded elsewhere in the genome. Similarly, *moaA*, which is involved in molybdopterin synthesis, a prerequisite for DMSO reductases, is found broadly among diverse taxa, so DIRM lacking the IRI associated *moaA* likely use molybdopterin synthesis genes elsewhere in the genome (Schwarz and Mendel, 2006). Lastly, many of the markers of horizontal transfer in *Denitromonas* sp. IR-12 also exists in *A. toluclasticum* sp. TC-10 including the *mer* and *cus* genes (8.5 and 15.2 kb upstream, respectively) (Juhas et al., 2009; Boyd and Barkay, 2012; Besaury et al., 2013). Thus, the presence of the IRI exclusively in TC-10, the variability of genes directly surrounding *idr*ABP_1_P_2_, and markers of horizontal transfer surrounding the IRI provide further evidence that DIR is a horizontally acquired metabolism.

## Discussion

This study cultured iodate reducing microorganisms from multiple freshwater and saltwater sources across the San Francisco Bay Area. Our survey yielded a new dissimilatory iodate respiring microorganism, *Aromatoleum toluclasticum* sp. TC-10, from freshwater creek sediment. This organism represents the third known DIRM in pure culture and the first isolated from a low-salinity environment (Farby, 2020). We demonstrated that *A. toluclasticum* sp. TC-10 couples IO_3_^–^ reduction to acetate oxidation to conserve energy for growth. TC-10 also possesses many of the same genomic and physiological features of the known DIRM *Denitromonas* sp. IR-12. Crucially, the lack of *cld* homologs in both *Denitromonas* sp. IR-12 and *A. toluclasticum* sp. TC-10 challenges previously proposed models suggesting a role for *cld* in DIR (Yamazaki et al., 2020). The paucity of *cld* among both validated and predicted DIRM further suggests that *cld* in the *Pseudomonas* sp. SCT genome is aleatory (Reyes-Umana et al., 2021). These observations are indicative of a parsimonious DIR model independent from *cld* and suggest that *Pseudomonas* sp. SCT reduces iodate like other DIRM. The *cld* in *Pseudomonas* sp. SCT is possibly a remnant of the composite transposon mobilizing the chlorate reduction island found in some *Pseudomonas* spp. previously (Clark et al., 2013). Future experiments exploring the protein biochemistry of the iodate reductase are needed to shed light on the intermediates of DIR.

This study also provides further evidence that the iodate reductase belongs to a phylogenetically distinct clade of molybdopterin oxidoreductases and represents a component part of a horizontally transferred composite genomic island containing the respiratory genes. We incidentally demonstrate the limitations of 16S rRNA gene sequencing in understanding the biology and ecology of environmental microorganisms (Woese et al., 1975; Achenbach and Coates, 2000; Federhen, 2012). Although 16S rRNA gene sequencing serves as a powerful tool for quickly identifying and classifying microorganisms, studies have long since noted that bacteria with identical 16S sequences can exhibit different gene content, nucleotide identity, and physiological capabilities (Achenbach and Coates, 2000). The similarity between both *Aromatoleum* isolates and modern sequencing technologies enabled a thorough comparison of the horizontally transferred IRI and its surrounding gene neighborhood. The iodate reductase A subunit (IdrA) clades with known DIRM, providing evidence that predicted DIRM in this group likely grow by DIR. We further validate the conserved synteny of the iodate reduction genes and show the limited conservation of genes surrounding the IRI. Genes involved in electron transfer (i.e., cyt C_551_) are missing in the TC-10 IRI, while molybdopterin cofactor synthesis genes are missing in the IR-12 IRI. Our observation suggests that DIRM lacking individual auxiliary components of the IRI likely use genomically encoded homologs. These results also affirm previous observations suggesting that genomes with conserved synteny at *idrABP_1_P_2_* are likely DIRM (Reyes-Umana et al., 2021). The diversity of genes surrounding the iodate respiration genes also suggests different evolutionary histories for the IRI. While no specific transfer mechanism was proposed due to a lack of clear evidence, we provide a detailed description of the IRI insertion site for future comparisons.

Ultimately, understanding the distribution, physiology, and diversity of DIRM depends on isolating and characterizing the physiology and genetics of new strains. Although we cannot rule out the possibility of marine origin from nearby sea spray, all *Aromatoleum* species to date have been isolated exclusively from pristine and contaminated freshwater sources (Rabus et al., 2019). Thus, our study expands the known distribution of the metabolism to coastal freshwater environments. Iodine deposited in these zones may be abundant enough for microorganisms to use it as a terminal electron acceptor. Therefore, future work surveying iodate removal should consider terrestrial sites in littoral zones with microbially mediated iodine biogeochemical cycling.

## Materials and Methods

### Media, Chemicals, and Culture Conditions

Anoxic enrichment cultures from freshwater environments were grown at 30°C in a freshwater minimal medium (FMM) containing 0.25 g NH4Cl, 0.40g NaH2PO4, 1.0 g Na2HPO4, 0.1 g KCl, and a vitamin and mineral mix described previously in Carlström et al. (2016). Enrichments cultures from marine environments used artificial pore medium (APM) previously described in Reyes-Umana et al. (2021). All media had a pH of 7.2, and oxygen was removed by boiling the media and cooling the media under an atmosphere of 100% N_2_. Media was subsequently dispensed into bottles or tubes under an atmosphere of 100% N_2_ and sealed with a butyl rubber stopper. Conditions containing iodate and acetate used the sodium salts of these compounds (Sigma Aldrich, United States). Cultures were grown aerobically on either FMM, R2A (HiMedia, United States), or R2A agar (BD Biosciences, United States). Growth of cultures was measured using a Thermo Scientific™ GENESYS™ 20 set at a wavelength of 600 nm. Growth cultures in Hungate tubes used a specially built adapter for measurement on the GENESYS™ 20. *Aromatoleum toluclasticum* sp. MF63 was procured from ATCC (ATCC 700605), recovered on Tryptic Soy Broth (BD Biosciences, United States). The 16S rRNA gene sequence and whole genome sequence are deposited in GenBank under accessions numbers OK665926 and PRJNA776029, respectively.

### Isolation of *Aromatoleum toluclasticum* sp. TC-10

Sediment from multiple locations across the San Francisco Bay Area (San Francisco, CA, United States) was collected. Sediment was added in triplicate in either anoxic APM or anoxic FMM when collected from marine or freshwater sources, respectively. After degassing under a 100% N_2_ atmosphere, all enrichments were amended with 2 mM iodate and 10 mM acetate and grown for 14 days. Iodate and acetate consumption was monitored, and microcosms showing total removal of iodate were plated on R2A agar. Single colonies were isolated on R2A agar, re-streaked, and confirmed by colony morphology, microscopic evaluation, and sequencing of the 16S rRNA gene. The microcosms containing riverbank sediment at Tunitas Creek (37°35′67.4″ N, −122°39′81.7″ W) near San Gregorio, California contained the isolate *Aromatoleum toluclasticum* sp. TC-10.

### Iodate, Acetate, and Iodide Quantification

A Dionex™ IonPac™ AS25 Anion Exchange Column was used on an ICS-1500 ion chromatography system (Thermo Fischer, United States) to measure iodate, iodide, and acetate in triplicate as described previously (Reyes-Umana et al., 2021). All samples were diluted at 1:20 in deionized water and loaded onto the autosampler for processing. A serial dilution starting at 1 mM of the standard molecule was used to generate the standard curve. Standards were linear over a range of 0.03 mM to 1 mM. Acetate peaks were consistently detected at 3.6 min, iodate peaks at 3.8 min, and iodide peaks at 11.5 min using a flow rate of 1 mL/min and a 36 mM NaOH eluent.

### Genome Sequencing, Comparative Genomics, and Phylogenetic Analysis

Genome sequencing and library preparation for both *A. toluclasticum* strains was performed by the Vincent J. Coates Genomics Sequencing Laboratory at UC Berkeley on an Illumina NovaSeq6000 using 150 paired-end reads (Illumina, United States). Reads passed FastQC v0.11 quality controls, were trimmed using Sickle v1.33, and assembled using SPAdes v3.9 (Bankevich et al., 2012). Prokka v1.14 using standard settings was used to generate genome annotations and the general feature format file (.gff), which was used to navigate and visualize the genome on the Artemis software^1^ (Seemann, 2014). The California regional map was drawn using Cartopy v0.19. The average nucleotide identity (ANI) was calculated using FastANI v1.32, and genome synteny and organization were evaluated using the nucmer function provided by Mummer v4.0 with default settings to produce a delta file and graphed using mummerplot with both the “layout” and “filter” tags present. Synteny and organization of contig 18 used similar methods but omitted the “layout” and “filter” tags to visualize inversions and rearrangements. Insertion site sequences were determined using the show-aligns function for contig 18 against the two neighboring contigs on the *A. toluclasticum* sp. MF63 reference genome using standard settings. The IslandViewer4 web interface and MGEfinder were used as orthologous methods to predict genomic islands from the draft genome (Bertelli et al., 2017; Durrant et al., 2020). Phylogeny and gene neighborhood analysis for IdrA was performed by redrawing the IdrA phylogenetic tree used in Reyes-Umana et al. with TC-10 (Reyes-Umana et al., 2021). Briefly, NasA, NasC, and NapA were used as outgroups, all sequences were aligned using MUSCLE v3.8, and the tree was drawn using standard settings on FastTree with the -boot tag set at 10,000 (Edgar, 2004; Price et al., 2010). The phylogenetic tree was visualized using iToL. The visualization of bootstrap values and gene neighborhoods was displayed using Adobe Illustrator (Letunic and Bork, 2016). Protein subfamilies were assigned by submitting sequences for analysis on Pfam 34.0. Clades were collapsed using the triangle visualization where the two side lengths are proportional to the node’s closest and farthest child leaves.

## Data Availability Statement

The datasets presented in this study can be found in online repositories. The names of the repository/repositories and accession number(s) can be found below: https://www.ncbi.nlm.nih.gov/genbank/, OK665926; https://www.ncbi.nlm.nih.gov/genbank/, PRJNA776029.

## Author Contributions

VRU and JDC designed research. VRU and JK performed all physiology experiments and measurements. VRU performed the comparative genomic analysis and phylogenetic analyses. VRU wrote the draft manuscript and created the figures with guidance from JDC. All authors contributed to data analysis, reviewed the manuscript, and approved of its publication.

## Conflict of Interest

The authors declare that the research was conducted in the absence of any commercial or financial relationships that could be construed as a potential conflict of interest.

## Publisher’s Note

All claims expressed in this article are solely those of the authors and do not necessarily represent those of their affiliated organizations, or those of the publisher, the editors and the reviewers. Any product that may be evaluated in this article, or claim that may be made by its manufacturer, is not guaranteed or endorsed by the publisher.

## Footnotes

1
http://sanger-pathogens.github.io
